# Influence of Framework Material on Biomechanical Performance of an All-on-4 Prosthesis Supported by Bendable Monoblock Implants

**DOI:** 10.3390/bioengineering13050581

**Published:** 2026-05-19

**Authors:** Esra Bilgi-Ozyetim, Ali Mushtaq Neamah Almaliki, Süleyman Çağatay Dayan, Onur Geçkili

**Affiliations:** 1 Department of Prosthodontics, Faculty of Dentistry, Istanbul Yeni Yuzyil University, Istanbul 34445, Türkiye; 2 Department of Prosthetic Technologies, College of Health & Medical Technology, AL-AYEN University (AUIQ), Thi-Qar 64001, Iraq; alialmaliki19951995@gmail.com; 3 Program of Dental Technicians, Istanbul University-Cerrahpaşa, Istanbul 34262, Türkiye; suleyman.dayan@iuc.edu.tr; 4 Department of Prosthodontics, Faculty of Dentistry, Istanbul University, Istanbul 34116, Türkiye; geckili@istanbul.edu.tr

**Keywords:** All-on-4 prosthesis, bendable monoblock implant, polyetheretherketone, polyetherketoneketone, glass fiber-reinforced polymer composite

## Abstract

The purpose of this study was to use the finite element analysis method to determine the influence of framework material on stresses in different parts of a model of an All-on-4 prosthesis supported by bendable monoblock implants. A three-dimensional solid model of an edentulous mandible was reconstructed from computed tomography data and segmented using 3DSlicer. Four bendable monoblock implants were positioned in accordance with the All-on-4 configuration. Screw-retained prostheses were modeled with the framework considered fabricated using one of five materials. These were cobalt–chromium (Co-Cr) alloy, titanium (Ti) alloy, polyetheretherketone (PEEK), polyetherketoneketone (PEKK), and a glass fiber-reinforced polymer composite (FRC) material. Four types of clinically relevant loads (300 N) were applied statically, namely, unilateral oblique, unilateral vertical, bilateral oblique, and bilateral vertical. Maximum and minimum principal stresses were determined in the cortical bone, and maximum von Mises stress was determined in each of the other parts of the model. Across most loading conditions, PEEK and PEKK showed higher stress values in the cortical bone and in the implants. In the screws, PEEK and PEKK also showed higher stress values, except in the anterior implant screws under bilateral loading conditions. In the framework, the highest stresses were obtained when a metal was the material of fabrication. Across all loading conditions, with FRC, the stress transfer was balanced. Thus, the prevent results suggest that FRC may be a suitable alternative to metallic materials for fabricating the framework of an All-on-4 prosthesis supported by bendable monoblock implants.

## 1. Introduction

Edentulism is a disabling condition that significantly affects patients’ functional and psychosocial well-being [1]. Although conventional complete dentures have been the primary treatment for more than a century, they are increasingly being replaced by implant-supported removable or fixed complete dentures, which provide superior comfort, function, and patient satisfaction [2,3].

Progressive resorption of the alveolar ridge can impose structural limitations that complicate implant placement. When bone volume is inadequate for implant-supported rehabilitation in the posterior regions, instead of advanced surgical techniques, such as various bone augmentation techniques, the use of short implants, or long cantilever restorations, implants can be tilted passing the maxillary sinus in maxilla or mental foramen in the mandible. In the completely edentulous jaw, positioning of four implants by tilting the most posterior ones between the mental foramina or maxillary sinus is a well-documented protocol demonstrating high clinical success rates [4,5]. Tilting the posterior implants reduces cantilever length and increases the bone–implant contact area, thereby improving stress distribution and minimizing peri-implant bone loading [6]. The concept of tilting posterior dental implants by up to 45° relative to the occlusal plane was first introduced by Malo and is known as the All-on-4 concept [7].

A rigid framework that splints these implants further enhances biomechanical stability by evenly transferring functional stresses [6]. Although angled multi-unit abutments are commonly used for the tilted distal implants in this concept, micro-gaps at the abutment–implant interface can harbor bacteria and toxins, predisposing patients to peri-implant inflammation [8,9]. Additionally, abutment screw loosening and fractures are frequent mechanical complications [10]. To eliminate such issues, bendable monoblock implants that integrate the abutment and the implant body into a single unit have been introduced [11]. Bendable monoblock implants offer significant advantages in All-on-4 prosthetic applications due to their inherent flexibility, which allows for better adaptation to the patient’s anatomical variations and reduces the risk of stress concentration at the implant interface [11].

The long-term success of implant-supported fixed complete dentures depends on understanding the underlying biomechanical principles of implantology to avoid bone overload and implant failure [12,13,14]. The geometry and material of the prosthetic framework play a crucial role in the distribution of mechanical stresses to the peri-implant bone [15]. The framework unites the implants into a single structure, enabling uniform transmission of functional loads [16].

Due to its low density, favorable mechanical properties, and biocompatibility, titanium (Ti) alloy is widely used to fabricate the framework [17]. However, Ti alloy frameworks have several drawbacks, including aesthetic limitations, casting difficulties, metallic taste, and incompatibility with imaging modalities [18]. Additionally, the high elastic modulus of Ti alloy (110 GPa) compared to that of bone (14 GPa) may induce stress shielding, which can potentially contribute to marginal bone loss and implant failure [18,19].

To address these concerns, polymeric frameworks, manufactured using computer-aided design/computer-aided manufacturing (CAD/CAM) technology, have been proposed as alternatives to frameworks fabricated using materials such as Ti alloy, cobalt-chromium (Co-Cr), and zirconia (ZrO_2_) [16,20,21,22]. High-performance polymers, such as polyetheretherketone (PEEK) and polyetherketoneketone (PEKK), offer several advantages, including low weight, low cost, and high shock absorption capacity. Their elastic modulus (~3.5 GPa for PEEK and 5.1 GPa for PEKK) is comparable to that of the human maxillary bone (cortical bone: ~13.7 GPa; trabecular bone: ~1.37 GPa) [23,24]. PEKK has a higher proportion of ketone groups, which provides superior thermal stability, greater potential for surface modification, and enhanced mechanical performance compared to PEEK [25]. Another promising alternative material is a glass fiber-reinforced polymer composite (FRC), which has an elastic modulus (~35 GPa) [26] lower than that of Co–Cr alloy (~218 GPa) [27] but higher than that of PEEK (3.5 GPa) [23].

Materials with high elastic moduli, such as metallic frameworks, transmit larger stresses to the bone-implant interface because they do not absorb occlusal shock [28,29]. In contrast, materials with lower moduli can act as stress breakers by dissipating occlusal forces and reducing stress concentration at the implant-bone interface [22]. Nonetheless, several studies suggest that a rigid framework may provide more favorable load distribution and reduce the risk of peri-implant overload [24,30,31].

The finite element analysis (FEA) is widely used in implant biomechanics due to its ability to simulate complex clinical conditions with high precision [32]. Given the increasing interest in high-performance polymers as alternatives to metals for fabricating the frameworks, evaluating their biomechanical behavior in implant-supported fixed complete dentures has become essential. However, the literature presents inconsistent findings regarding the effect of framework material rigidity on stress distribution in implant-supported prostheses. While some studies [33,34] have shown that polymer-based materials with lower elastic modulus increase stress transfer to the implant and the surrounding bone, others [35,36] have reported reduced stress levels in the peri-implant bone associated with the use of less rigid or semi-rigid materials, which may provide more uniform load distribution.

In addition, limited information is available regarding the biomechanical performance of different framework materials in implant-supported fixed complete dentures supported by bendable monoblock implants following the All-on-4 concept. Because monoblock implants eliminate the conventional implant–abutment interface, the mechanical behavior of implant-supported fixed complete dentures supported by such implants may differ from that of conventional restorations. Therefore, evaluating the effect of framework material selection in such configurations is clinically relevant. The purpose of the present study was to use FEA to determine the influence of framework material on stresses in the cortical bone, the bendable monoblock implant, screws, and the framework in a three-dimensional (3D) model that comprised a fixed complete denture supported by four implants arranged in a configuration that follows the All-on-4 concept. The framework materials used were Ti alloy, Co-Cr, PEEK, PEKK, and FRC. The null hypothesis was that the framework material would not affect the stress distribution patterns in either the bone or the bendable implants.

## 2. Materials and Methods

The study protocol was conducted in accordance with the principles of the Declaration of Helsinki and was approved by the Istanbul Yeni Yuzyil University Science and Health Sciences Research Ethics Committee (Approval No. 2023/10-1100, Approval Date: 2 October 2023).

**Figure 1:**
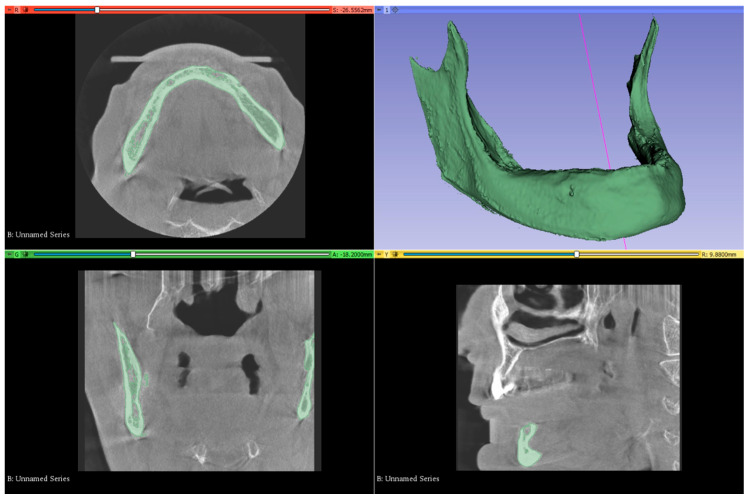
Segmentation of mandibular bone from computed tomography (CT) data and three-dimensional reconstruction using 3DSlicer software.

The generation of the 3D mesh, its transformation into a mathematically optimized solid model, the construction of the finite element models, and the FEA were all performed on an HP workstation (Intel Xeon E-2286, 2.40 GHz, 64 GB ECC memory). Bone structure was modeled using tomographic data obtained from the Visible Human Project (The National Library of Medicine, FACT SHEETS Office of Communications and Public Liaison National Library of Medicine, Bethesda, MD, USA), a publicly accessible and fully anonymized anatomical dataset, and, thus, requiring individual informed consent was not required. Bone structures were segmented from computed tomography (CT) data using 3DSlicer software (version 5.10.0). STL models were extracted from CT data via 3DSlicer (Figure 1). Reverse engineering and 3D CAD modeling were performed using ANSYS SpaceClaim (Version 22.0), while model preparation and optimized meshing were performed in ANSYS Workbench. The finite element simulations were solved using the LS-DYNA solver.

### 2.1. Modeling of Cortical and Trabecular Bone

**Figure 2:**
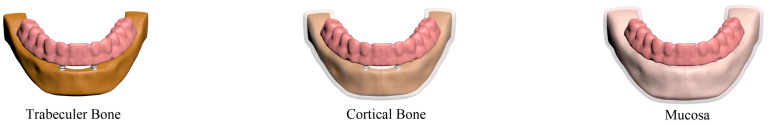
Segmented and reconstructed models of mandibular trabecular bone, cortical bone, and mucosa.

The mandibular bone model used in the study was obtained from the CT scan of an edentulous adult. Dataset was obtained from Visible Human Project. The subject was female; however, age information was not available. The CT scan was reconstructed with a slice thickness of 0.1 mm, and the resulting data were imported in DICOM (.dcm) format into 3DSlicer software. Based on appropriate Hounsfield Unit (HU) thresholds, the DICOM data were segmented to produce three-dimensional representations of the bone structures (Threshold range: 426.50–3193.04). Unnecessary regions and artifacts were removed using the “Erase” and “Scissors” tools. These models were then exported in STL (.stl) format. These 3D models were imported into ANSYS SpaceClaim, and a 2 mm cortical bone layer was created by applying a 2 mm offset to the mandibular model. The internal surface of the cortical shell was used as a reference to generate the trabecular bone model (Figure 2). All models were positioned in the 3D coordinate space using ANSYS SpaceClaim to ensure anatomical alignment, completing the modeling phase.

### 2.2. Model Construction

**Figure 3:**
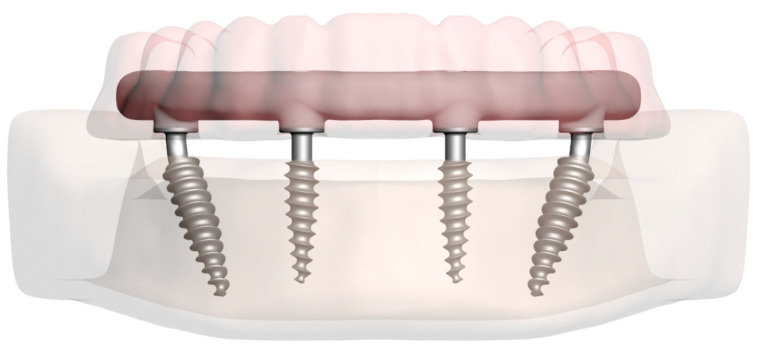
Three-dimensional finite element model of the All-on-4 concept.

Three-dimensional (3D) CAD models of bendable monoblock implants (Mode Provo-S, Mode Implant/Mode Medikal San. Tic. Ltd. Şti., İstanbul, Türkiye), were obtained from the manufacturer (Mode Implant/Mode Medikal San. Tic. Ltd. Şti., İstanbul, Türkiye). Two anterior implants (3.5 mm in diameter and 12 mm in length) were placed vertically in the lateral incisor regions, while two posterior implants (4.0 mm in diameter and 15 mm in length) were positioned at a 30° relative to the occlusal plane [37] in the second premolar regions (Figure 3).

**Figure 4:**
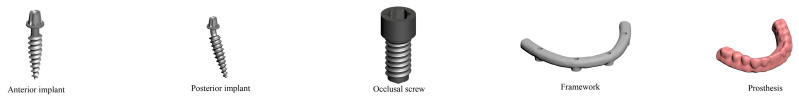
3D models of the implants, occlusal screw, framework, and prosthesis.

Occlusal screws were modeled in ANSYS SpaceClaim software based on the dimensions specified in the manufacturer’s catalog. The framework and prosthetic structures were also designed using ANSYS SpaceClaim. To ensure proper load transfer between the components, mesh compatibility adjustments were made in ANSYS Workbench (Figure 4).

**Figure 5:**
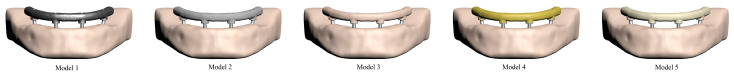
Comparative models representing five different framework materials used in the biomechanical analysis: Model 1—cobalt-chromium (Co-Cr), Model 2—Ti alloy, Model 3—polyetheretherketone (PEEK), Model 4—polyetherketoneketone (PEKK), Model 5—fiber-reinforced composite (FRC).

Implant-supported screw-retained fixed complete denture with frameworks made of Co-Cr, Ti alloy, PEEK, PEKK, or FRC were connected to implants via occlusal screws, and separate models were constructed for each framework material. Each prosthetic superstructure consisted of 12 teeth. In the polymer-based group (PEEK, PEKK, and FRC), the superstructure was veneered with light-cure composite resin, while in the metal groups (Co-Cr and Ti alloy), porcelain was used as the veneering material (Figure 5 and Table 1).

The bar structure was designed with a width of 5 mm, a height of 5 mm, and a cantilever length of 10 mm [35,37,38]. In the final prosthetic configuration, the cantilever was extended to 15 mm [39], and the vertical distance between the prosthesis and the mucosal surface was also set at 15 mm [35].

### 2.3. Generation of Mathematical Models

**Figure 6:**
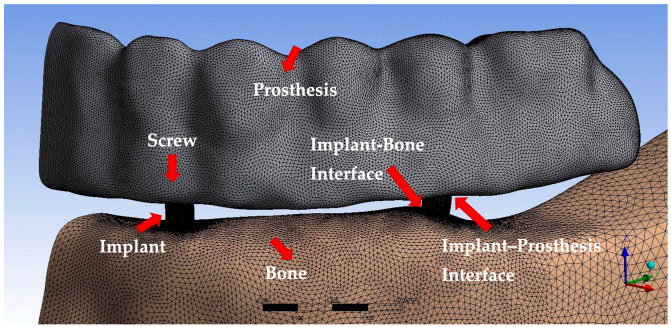
The meshed prosthesis, implant, screws, the supporting bone structures, and the interfaces in the model.

Mathematical models were generated by discretizing the geometric structures into small, simple elements in a process known as meshing (Figure 6). The three-dimensional geometrical models developed in ANSYS SpaceClaim were imported into ANSYS Workbench and converted into numerical models suitable for FEA. In the creation of the mathematical models (mesh structures), tria (triangular) mesh sizes ranging from 0.05 to 0.25 mm (highly refined) were used. After the surface meshes of all models were generated using tria, the solid meshes of the objects were created using tetrahedral (regular four-sided) solid mesh elements. The posterior and inferior regions of the bone were fixed in all three spatial directions. All components were defined using bonded contact conditions, thereby assuming perfect osseointegration at the bone–implant interface. Subsequently, these models were exported to the LS-DYNA solver for computational analysis. Linear elastic material behavior was assumed for all analyses; as such, each material was defined by its elastic modulus and Poisson’s ratio. The mechanical properties of all materials were obtained from previously published studies [23,24,26,27,37,40,41,42] (Table 2).

### 2.4. Mesh Convergence Test

A mesh convergence analysis was performed to ensure the reliability and accuracy of the finite element model used in the biomechanical analysis. The primary objective was to determine an appropriate mesh density that would achieve a relative error below 3% while maintaining a balance between computational efficiency and solution accuracy.

Accordingly, a series of finite element meshes with varying element sizes, ranging from coarse to fine, were generated. To ensure consistency, each mesh was analyzed under identical loading (Table 3) and boundary conditions, which are described in detail in the following section. For the mesh convergence analysis, the maximum von Mises stress in the implant was used as the evaluation parameter. The results obtained from successive mesh refinements were compared, and variations in the evaluation metric were observed. The relative error in the stress obtained between use of consecutive meshes was calculated using the following equation:

Relative Error (%) = [(Value (Updated Mesh) − Value (Previous Mesh))/Value (Updated Mesh)] × 100

During model preparation, triangular 2D and tetrahedral 3D meshes were used. These mesh types are considered suitable for modeling complex geometries and curved surfaces, such as bone structures. Mesh quality was evaluated for all models, and elements with a skewness value greater than 80° or a minimum element size below 0.001 were reviewed. Meshes did not conform to these criteria were appropriately corrected and included in the analysis.

### 2.5. Loading and Boundary Conditions

**Figure 7:**
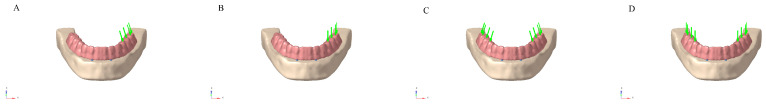
The types of loading applied to the model. (**A**) Unilateral oblique load; (**B**) Unilateral vertical load; (**C**) Bilateral oblique load; (**D**) Bilateral vertical load.

In fixed prostheses following the “All-on-4” concept, the average occlusal force in the premolar and molar regions has been reported to be approximately 200–300 N [43]. In the present study, to simulate different masticatory conditions, loads were applied to all models under unilateral oblique, unilateral vertical, bilateral oblique, and bilateral vertical loads scenarios. In the oblique loading condition, a 300 N force was applied at a 45° angle from the lingual to the buccal direction on the buccal cusps of the first premolar, second premolar, and first molar teeth. In the vertical loading condition, a 300 N force was applied perpendicularly to the same regions (Figure 7). The occlusal load was distributed on the three posterior teeth.

**Figure 8:**
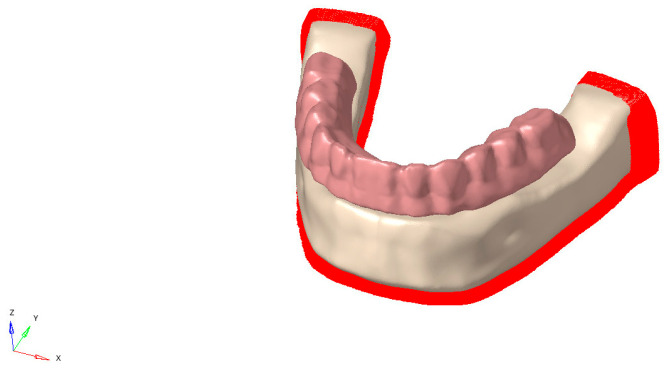
Boundary conditions of the finite element model, showing the fixed regions of the mandibular bone.

The applied forces were distributed over adjacent nodes to avoid stress singularities at the loading regions. All models were constrained at the posterior and inferior regions of the mandible by fixing the corresponding nodes and restricting all degrees of freedom in the X, Y, and Z directions. Additionally, symmetric boundary conditions were applied to all model components in the Y–Z plane, normal to the *X*-axis (Figure 8). Under these loading and boundary conditions, a total of 20 linear static analyses were conducted across the five framework material models (Table 4).

### 2.6. Stress Distribution Analysis

In this study, stress criteria were selected based on the mechanical behavior of each material. All prosthetic components (implants and screws) and framework structures in the “All-on-4” system were analyzed using von Mises equivalent stress, which is appropriate for ductile materials. For cortical bone, the maximum (tensile) (σ_max_) and minimum (compressive) (σ_min_) principal stresses were calculated according to the failure theory of maximum principal stress. This method was preferred because bone exhibits lower tolerance to tensile loads than to compressive loads and exhibits behavior similar to brittle materials [23].

## 3. Results

### 3.1. Maximum Principal Stresses (σ_max_) and Minimum Principal Stress (σ_min_) in Cortical Bone

**Figure 9:**
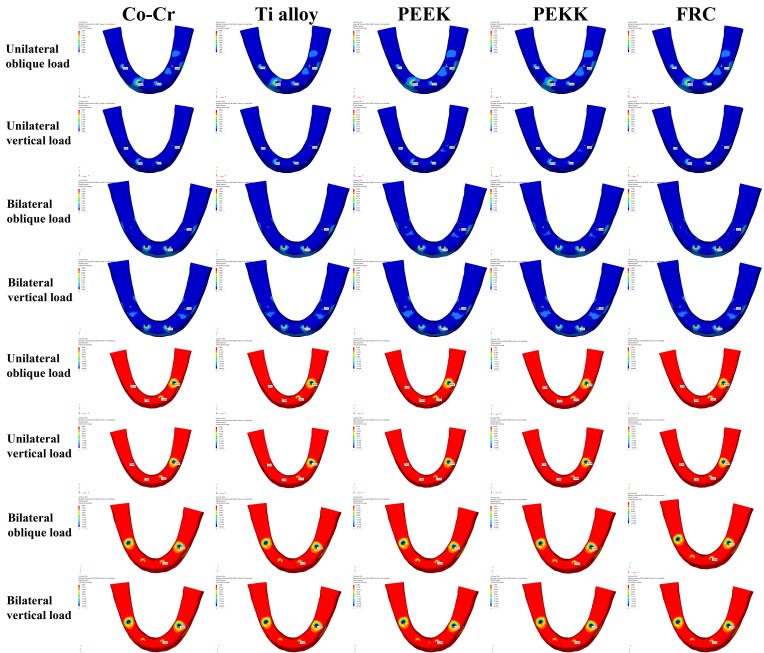
Maps of σ_max_ (in blue) and σ_min_ (in red) in the cortical bone under unilateral oblique, unilateral vertical, bilateral oblique, and bilateral vertical loads.

These results are shown in Figure 9 and Table 5. In the anterior region, the highest σ_max_ was observed at the right anterior site in the PEEK group under unilateral oblique loading (23.156 MPa), whereas the lowest value was recorded at the left anterior site in the Co–Cr group under unilateral vertical loading (5.557 MPa). In the posterior region, the highest σ_max_ was observed at the left posterior site in the PEEK group under unilateral oblique loading (16.368 MPa), whereas the lowest value was recorded at the right posterior site in the PEEK group under unilateral vertical loading (0.840 MPa).

In cortical bone, higher σ_min_ values were observed in the posterior region compared with the corresponding anterior region on the same side across all loading conditions. In the anterior region, the highest σ_min_ was also observed in the PEEK group under unilateral oblique loading at the left site (−23.434 MPa), while the lowest value was obtained in the Co–Cr group under unilateral vertical loading at the right site (−2.188 MPa). In the posterior region, the highest σ_min_ was observed in the PEEK group under unilateral oblique loading at the left site (−94.467 MPa), whereas the lowest value was recorded in the same group under unilateral vertical loading at the right site (−3.635 MPa).

Across most unilateral loading conditions and all bilateral loading conditions, the framework materials exhibited a consistent ranking of σ_max_ and σ_min_ values: PEEK > PEKK > FRC > Ti alloy > Co–Cr.

### 3.2. Maximum Von Mises Stress in Implant

**Figure 10:**
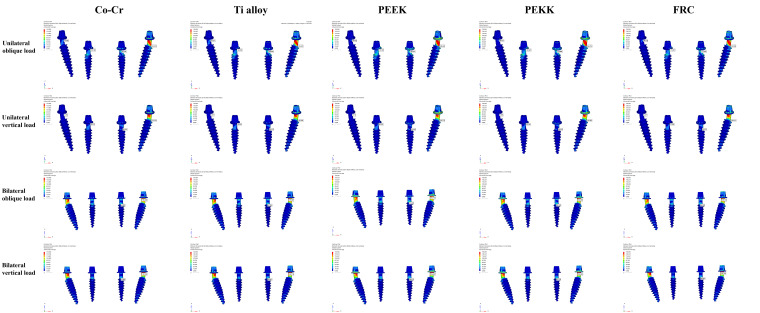
Color-coded maps of the maximum von Mises stress distributions in the implant structures under unilateral oblique, unilateral vertical, bilateral oblique, and bilateral vertical loads.

These results are shown in Figure 10 and Table 6. The highest maximum von Mises stress was observed in the PEEK group under unilateral oblique loading at the left posterior site (239.365 MPa), whereas the lowest value was recorded in the same group under unilateral vertical loading at the right posterior site (3.176 MPa). In the anterior region, the highest maximum von Mises stress was observed at the right anterior site in the PEEK group under unilateral oblique loading (131.667 MPa), whereas the lowest value was recorded at the right anterior site in the Co–Cr framework under unilateral vertical loading (35.571 MPa). Under unilateral oblique and vertical loading conditions, posterior implants at the right site exhibited markedly lower maximum von Mises stress values compared to the corresponding anterior implants. Under unilateral loading, vertical loading yielded lower maximum von Mises stress values than oblique loading across all implants. Under bilateral oblique and vertical loading conditions, posterior implants exhibited higher maximum von Mises stress values than anterior implants.

### 3.3. Maximum Von Mises Stress in Screws

**Figure 11:**
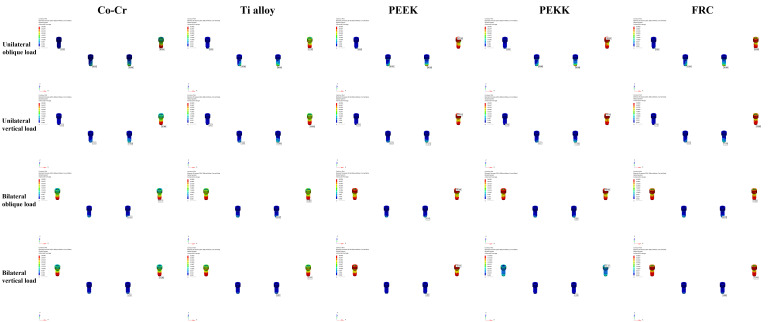
Color-coded maps of the maximum von Mises stress distributions in the screws, under unilateral oblique, unilateral vertical, bilateral oblique, and bilateral vertical loads.

These results are shown in Figure 11 and Table 7. The highest maximum von Mises stress was observed in the PEEK group under unilateral oblique loading at the left posterior implant site (112.104 MPa), whereas the lowest value was recorded in the Co-Cr group under unilateral vertical loading at the right posterior implant site (3.022 MPa). In the anterior implant region, the highest stress value was observed in the PEEK group under unilateral oblique loading at the right anterior site (26.375 MPa), whereas the lowest value was recorded in the PEEK group under bilateral vertical loading (3.881 MPa). Under bilateral oblique and vertical loading, the posterior implant showed significantly higher maximum von Mises stress values than the anterior implant for all framework materials. Under unilateral oblique and vertical loading conditions (except for the left posterior implant under unilateral vertical loading), the highest maximum von Mises stress values were observed in the PEEK group at both anterior and posterior implants on the right and left sides. Under bilateral oblique and vertical loading conditions, the highest stress values were observed in the PEEK group and the lowest in the Co–Cr group at the posterior implant, whereas at the anterior implant, the highest stress values were observed in the metal framework groups and the lowest in the PEEK group.

### 3.4. Maximum Von Mises Stress in the Framework

**Figure 12:**
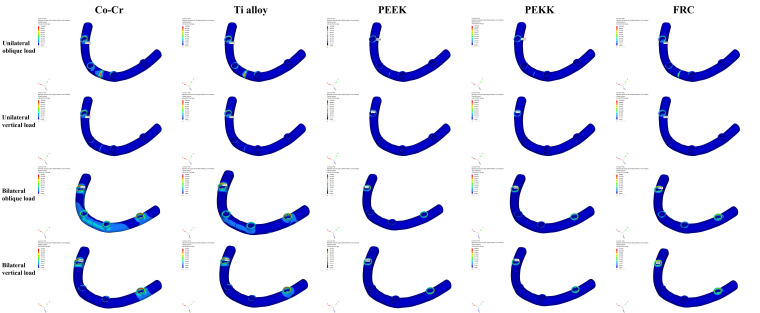
Color-coded maps of von Mises stress distributions in the prosthetic framework under unilateral oblique, unilateral vertical, bilateral oblique, and bilateral vertical loads.

These results are shown in Figure 12 and Table 8. The highest maximum von Mises stress was observed in the Co–Cr framework under unilateral oblique loading (134.336 MPa), whereas the lowest value was recorded in the PEEK framework under unilateral vertical loading (32.195 MPa). Across all loading conditions, the highest maximum von Mises stress values were observed in the Co–Cr group, followed by Ti alloy, FRC, PEKK, and the lowest in the PEEK, with metal frameworks (Co–Cr and Ti alloy) exhibiting higher maximum von Mises stress values than polymer-based frameworks. In all framework groups, the highest maximum von Mises stress values were observed under unilateral oblique loading conditions. Midline (symphysis) stress accumulation was observed in Co–Cr and Ti alloy frameworks under unilateral oblique, unilateral vertical, and bilateral oblique loading, whereas polymer-based frameworks (PEEK, PEKK, and FRC) exhibited midline stress only under unilateral oblique loading.

## 4. Discussion

Understanding stress distribution at the implant-bone interface is essential for ensuring the long-term stability of dental implants. The magnitude of stress transmitted from a prosthetic framework to the surrounding bone is influenced by the elastic modulus of the framework material [44]. While rigid metallic frameworks remain the gold standard for implant-supported fixed complete dentures, there is limited information regarding the biomechanical behavior of high-performance polymer-based materials, particularly when used with bendable monoblock implants. Most current investigations are restricted to a single material group or standardized, simplified occlusal scenarios [45,46]. This study aimed to clarify the biomechanical performance of FRC, under unilateral oblique, unilateral vertical, bilateral oblique, and bilateral vertical loading conditions, in comparison with PEEK, PEKK, and conventional metallic alloys. The results indicated that polymer-based frameworks with lower elastic moduli transmitted noticeably higher stresses to the cortical bone and implants. As such, the null hypothesis of the study was rejected.

The results demonstrated that framework material and loading configuration significantly influenced stress distribution across cortical bone, implants, screws, and framework structures. PEEK and PEKK frameworks exhibited the lowest stress levels within the framework, indicating effective stress-absorbing behavior; however, this was accompanied by increased stress concentrations in cortical bone, implants, and screws, reflecting load transfer to the supporting components. This behavior is attributed to the low elastic modulus of these materials, which allows greater framework deflection and shifts stress toward the implant–abutment interface. While flexible frameworks reduce internal stress, they may increase stress at critical interfaces, potentially predisposing to screw loosening or abutment fatigue [47,48]. Conversely, Co–Cr and Ti alloy frameworks exhibited the highest stress values within the framework due to their high rigidity, while transmitting relatively lower stress to cortical bone, implants, and screws. FRC demonstrated an intermediate biomechanical response, providing a balanced stress distribution between structural rigidity and load transfer to surrounding tissues. This intermediate behavior can be explained by the materials elastic modulus (approximately 35 GPa), which lies between low-modulus polymers (PEEK: ~3.5 GPa; PEKK: ~5.1 GPa) and high-modulus alloys (Ti alloy: ~110 GPa; Co–Cr: ~218 GPa), which allows partial stress absorption while limiting excessive stress transfer to surrounding structures. In addition, the loading configuration played a critical role in determining the magnitude of stress. Oblique loading consistently produced higher stress values than vertical loading across all components, likely due to the presence of lateral force and bending moment. Furthermore, posterior implant regions exhibited more pronounced stress variations, particularly under unilateral loading conditions, which may be associated with cantilever effects and asymmetric load distribution.

Some of the trends in the results in the present work are the same as those reported in the relevant literature studies. Dayan and Geckili [47] reported that PEEK and PEKK frameworks exhibited lower internal stresses while transferring greater stress to surrounding bone and implant components. Güzelce [26] reported that Co-Cr frameworks displayed higher internal stress, resulting in lower stress values in the supporting bone and implants. Villefort et al. [49] compared PEKK and PEEK and found that PEKK frameworks exhibited lower stress concentrations at the prosthetic screw than PEEK, suggesting a reduced risk of screw loosening. Meghna et al. [48] reported that the highest stress values in the framework were observed in the Co-Cr group, whereas the lowest stress values were observed in the PEEK group. However, unlike the findings of the present study, they also reported that the lowest stress values in the bone were observed in the PEEK group. de Araújo Nobre et al. [50] reported that the use of PEEK frameworks and hybrid PEEK-acrylic resin prostheses in All-on-4 restorations reduces marginal bone loss and improved long-term biological outcomes, which was attributed to the shock-absorbing properties of PEEK. Similarly, Chen et al. [40] found that the use of a PEEK framework provided more favorable stress distribution in the jawbone due to its lower elastic modulus. Cabbarova et al. [46] reported that materials with low elastic modulus (PEEK and PEKK) increased stress transmission to peri-implant bone and connection components, whereas rigid materials such as Ti alloy and zirconia provided a more balanced load distribution with lower stress concentrations. In addition, FRC frameworks remained within clinically acceptable biomechanical limits. Similarly, Lahoud et al. [51] reported that FRC exhibited higher fracture resistance than zirconia, indicating its potential as a reliable alternative for full-arch rehabilitations. Demirci et al. [52] also found that FRC demonstrated significantly greater fracture loads compared to PEEK and PEKK, while Franco et al. [53] showed that FRC frameworks achieved a more uniform occlusal load distribution around implants compared to rigid metallic structures. In line with these findings, the present study demonstrated that the FRC framework exhibited lower σ_max_ and σ_min_ stress values in cortical bone compared to polymer-based framework and higher values compared to metal-based framework under bilateral and unilateral loading conditions, revealing that the FRC framework demonstrated a balanced biomechanical response. In the present study, conventional multi-unit abutments were replaced with bendable monoblock implants specifically designed for compatibility with the All-on-4 configuration. Monoblock implants eliminate both the abutment screw and the microgap at the implant-abutment interface, which can reduce stress concentrations in the connection region. Zincir and Parlar [3] reported that monoblock implants resulted in lower stress levels around the implant and peri-implant bone compared to traditional systems. The results of the current study further support these observations, indicating that monoblock configurations promote a more uniform stress distribution, potentially minimizing marginal bone loss and enhancing long-term biomechanical stability. The maximum von Mises stress values in metallic frameworks (Co-Cr and Ti alloy) were primarily concentrated within the framework structure but remained well below the yield strengths of these materials [54,55]. In our study, the maximum tensile stress (σ_max_) values in cortical bone remained below the allowable limit of 66 MPa across all groups and loading conditions [35]. Notably, under certain loading scenarios, σ_max_ values in the anterior region surpassed those in the posterior, which has clinical relevance given the limited tensile strength of cortical bone. However, since all values remained within safe limits, we conclude that no structural risks were present. When examining minimum compressive stress (σ_min_) values, relatively low stress levels were observed in the anterior regions, indicating that loads were primarily transferred to the posterior implants.

The findings of this study contribute to the evolving understanding of stress distribution at the implant-bone interface, particularly regarding the comparative performance of PEEK, PEKK, and FRC frameworks versus metallic frameworks. The observed differences in stress transfer mechanisms underscore the importance of material selection in optimizing implant design and enhancing the longevity of dental restorations. Further research is essential to explore the long-term implications of these materials in clinical practice. To enhance the clinical relevance of future investigations, additional in vitro and in vivo studies are necessary. These studies should examine the hybrid biomechanical performance of FRC frameworks under dynamic loading conditions while considering varying implant angulations, interface configurations, and load application types.

This study has several limitations. The finite element model assumed that all materials were isotropic, homogeneous, and linearly elastic, which does not fully represent the complex biomechanical behavior of biological tissues. Additionally, the reliance on anatomical data from a single patient restricts the generalizability of the results. The study utilized static loading conditions, failing to account for the dynamic nature of actual chewing activities. The long-term performance of materials under cyclic loading and potential fatigue failure were not assessed. The study overlooked biological factors that could influence implant success, such as individual healing responses and bone remodeling processes; in addition, different materials were used as the veneer agent on the superstructure (the 12 teeth), which may influence stress distribution patterns. Moreover, different framework materials not included in this study may affect biomechanical behavior, and further studies evaluating different material combinations are needed. Another limitation is that important biomechanical responses, such as displacement and micromotion of implants and prosthetic components, were not analyzed. Lastly, the results of this study are specific to a fixed set of parameters, including implant design, posterior implant angulation, and cantilever length, and variations in these factors may significantly influence stress distribution patterns.

## 5. Conclusions

Within the limitations of this study:

1. In cortical bone, the highest σ_max_ and σ_min_ values were observed in the PEEK group under unilateral and bilateral loading conditions, while the lowest values were observed in Co–Cr (except for the right posterior sites under unilateral oblique and vertical loads).
2. In the implants, the highest maximum von Mises stress values were observed in the PEEK group under unilateral loading (except for the right posterior implants under oblique and vertical loads) and under vertical loading.
3. In the screws, the highest maximum von Mises stress values were observed in the PEEK group, under most of the loading conditions.
4. In the framework, the highest maximum von Mises stress values were observed in Co–Cr group, while the lowest values were observed in the PEEK group across all loading conditions.

Balanced stress distributions were observed in the FRC group, with lower stresses in the cortical bone and supporting components than in polymer-based frameworks, and lower internal stress concentrations than in metallic frameworks. These findings suggest that FRC could serve as a promising alternative to conventional frameworks utilized in All-on-4 prostheses supported by bendable monoblock implants. Nonetheless, it is essential to conduct further biomechanical and clinical studies to substantiate these preliminary observations and fully assess the viability of FRC in this context.

## Figures and Tables

**Table 1:** Study models.

Model	Framework and Superstructure Materials
**Model 1**	Co-Cr bar with metal porcelain superstructure
**Model 2**	Ti alloy bar with metal porcelain superstructure
**Model 3**	PEEK bar with composite resin superstructure
**Model 4**	PEKK bar with composite resin superstructure
**Model 5**	FRC bar with composite resin superstructure

**Table 2:** Quantitative model information.

Material	Elastic Modulus (MPa)	Poisson’s Ratio (ν)	Source
**Cortical Bone**	13,700	0.3	[37]
**Trabecular Bone**	1370	0.3	[24]
**Ti alloy (implant screw)**	110,000	0.35	[40]
**Co-Cr (framework)**	218,000	0.33	[27]
**Ti alloy (framework)**	110,000	0.35	[41]
**PEEK (framework)**	3500	0.4	[23]
**PEKK (framework)**	5100	0.4	[24]
FRC * (framework)	35,000	0.4	[26]
**Porcelain**	82,800	0.35	[42]

* 45 vol./vol.% glass fibers. Fibers were densely arranged in a multilayer, parallel, and bidirectional orientation within the polymer matrix.

**Table 3:** Mesh convergence test results based on maximum von Mises stress.

Model	Mesh Size (mm)	Maximum Von Mises Stress in Implant (MPa)	Relative Error (%)
Bilateral Oblique PEEK	0.4	150.260	-
	0.3	167.571	10.33
	0.2	179.048	6.41
	0.1	183.752	2.56

**Table 4:** Mesh characteristics of the finite element models.

	Total # of Elements	Total # of Nodes
**Model 1 Bilateral**	2,685,390	651,580
**Model 2 Unilateral**	5,370,780	1,303,158

**Table 5:** Maximum principal stress (σ_max_) and minimum principal stress (σ_min_) in the cortical bone under unilateral oblique, unilateral vertical, bilateral oblique, and bilateral vertical loads.

		σ_max_				σ_min_			
		Right Anterior	Right Posterior	Left Anterior	Left Posterior	Right Anterior	Right Posterior	Left Anterior	Left Posterior
**Unilaterale oblique load**	**Co-Cr**	**19.867**	**12.012**	**9.846**	**15.288**	**−7.220**	**−10,379**	**−19.402**	**−87.168**
	**Ti alloy**	20.321	11.089	9.967	15.467	−7.548	−9.187	−19.552	−88.159
	**PEEK**	**23.156**	**7.778**	**10.675**	**16.368**	**−10.773**	**−4.94**	**−23.434**	**−94.467**
	**PEKK**	22.706	8.005	10.621	16.263	−10.273	−6.105	−20.550	−93.509
	**FRC**	21.090	9.675	10.179	15.757	−8.292	−7.378	−19.651	−89.875
**Unilateral vertical load**	**Co-Cr**	9.881	**1.541**	**5.557**	**12.48**	**−2.188**	**−4.883**	**−12.387**	**−72.052**
	**Ti alloy**	**9.879**	1.375	5.636	12.513	−2.356	−4.603	−12.786	−72.38
	**PEEK**	**10.401**	**0.840**	5.819	**12.685**	**−3.581**	**−3.635**	**−14.503**	**−75.230**
	**PEKK**	10.295	0.876	**5.849**	12.665	−3.393	−3.713	−13.099	−74.669
	**FRC**	9.947	1.139	5.740	12.568	−2.657	−4.207	−12.851	−73.015
		**Anterior**		**Posterior**		**Anterior**		**Posterior**	
**Bilateral oblique load**	**Co-Cr**	**13.459**		**12.410**		**−12.645**		**−76.208**	
	**Ti alloy**	13.654		12.420		−12.797		−76.587	
	**PEEK**	**14.585**		**12.506**		**−13.485**		**−79.657**	
	**PEKK**	14.292		12.488		−13.412		−79.046	
	**FRC**	13.916		12.437		−13.019		−77.215	
**Bilateral vertical load**	**Co-Cr**	**11.483**		**12.791**		**−11.007**		**−76.847**	
	**Ti alloy**	11.523		12.797		−11.025		−77.069	
	**PEEK**	**12.157**		**12.882**		**−11.502**		**−79.758**	
	**PEKK**	12.084		12.863		−11.440		−79.189	
	**FRC**	11.646		12.812		−11.113		−77.568	

**Table 6:** The maximum von Mises stress (in MPa) values in the anterior/posterior implants under unilateral oblique, unilateral vertical, bilateral oblique, and bilateral vertical loads.

		Oblique Loading				Vertical Loading			
		Right Anterior Implant	Right Posterior Implant	Left Anterior Implant	Left Posterior Implant	Right Anterior Implant	Right Posterior Implant	Left Anterior Implant	Left Posterior Implant
**Unilateral load**	Co-Cr	**100.786**	**40.588**	**94.178**	**194.519**	**35.571**	**5.13**	62.422	**125.519**
	Ti alloy	106.184	39.157	100.935	201.425	36.455	4.877	**62.375**	126.406
	PEEK	**131.667**	38.493	**128.757**	**239.365**	**40.222**	**3.176**	**64.494**	**132.779**
	PEKK	130.326	38.437	126.684	234.218	40.158	3.308	64.07	131.896
	FRC	115.835	**38.128**	111.649	212.82	37.978	4.277	62.572	128.143
		**Anterior Implant**		**Posterior Implant**		**Anterior Implant**		**Posterior Implant**	
**Bilateral load**	Co-Cr	**66.891**		180.48		**61.685**		182.726	
	Ti alloy	67.78		179.887		62.052		182.176	
	PEEK	**75.48**		**183.752**		**68.476**		**186.967**	
	PEKK	74.19		182.008		67.376		184.894	
	FRC	69.506		**178.745**		63.234		**181.256**	

**Table 7:** Maximum von Mises stress (in MPa) values in the screws under unilateral oblique, unilateral vertical, bilateral oblique, and bilateral vertical loads.

		Oblique Loading				Vertical Loading			
		Right Anterior Screw	Right Posterior Screw	Left Anterior Screw	Left Posterior Screw	Right Anterior Screw	Right Posterior Screw	Left Anterior Screw	Left Posterior Screw
**Unilateral load**	**Co-Cr**	**18.537**	6.149	**19.444**	**56.119**	**7.673**	**3.022**	11.429	**51.369**
	**Ti alloy**	19.728	**5.975**	20.297	57.765	7.889	3.047	11.454	52.919
	**PEEK**	**26.375**	**6.424**	**26.120**	**112.104**	**9.475**	**3.545**	**11.414**	**106.045**
	**PEKK**	25.680	6.372	25.269	104.521	9.271	3.460	11.498	99.249
	**FRC**	21.877	5.979	21.929	60.968	8.334	3.135	**11.509**	55.888
		**Anterior Screw**		**Posterior Screw**		**Anterior Screw**		**Posterior Screw**	
**Bilateral load**	**Co-Cr**	10.152		**49.417**		**5.103**		**51.280**	
	**Ti alloy**	**10.397**		50.861		5.092		52.761	
	**PEEK**	**9.244**		**107.793**		**3.881**		**108.950**	
	**PEKK**	9.601		100.605		4.196		101.767	
	**FRC**	10.370		53.582		4.989		55.497	

**Table 8:** The maximum von Mises stress (in MPa) values in the prosthetic framework under unilateral oblique, unilateral vertical, bilateral oblique, and bilateral vertical loads.

	Bilateral		Unilateral	
	Oblique	Vertical	Oblique	Vertical
**Co-Cr**	**79.2**	**78.046**	**134.336**	**114.562**
**Ti alloy**	65.152	64.308	116.074	99.86
**PEEK**	**33.136**	**35.494**	**37.868**	**32.195**
**PEKK**	**33.136**	37.811	39.549	32.56
**FRC**	38.091	38.213	69.893	62.056

## Data Availability

The dataset is available from the corresponding author upon reasonable request.

## Abbreviations

CAD/CAM	Computer-aided design/computer-aided manufacturing
Co-Cr	Cobalt-chromium
CT	Computed tomography
FEA	Finite element analysis
FRC	Fiber-reinforced composite
HU	Hounsfield Unit
PEEK	Polyetheretherketone
PEKK	Polyetherketoneketone
σ_max_	Maximum principal stress
σ_min_	Minimum principal stress
ZrO_2_	Zirconia
3D	Three-dimensional
